# The complete mitochondrial genome of *Nais communis* Piguet, 1906 (Annelida; Clitellata; Naididae)

**DOI:** 10.1080/23802359.2021.1944378

**Published:** 2022-01-17

**Authors:** Jeounghee Lee, Jongwoo Jung

**Affiliations:** a Interdisciplinary Program of Ecocreative, Ewha Womans University, Seoul, Korea; b Department of Science Education, Ewha Womans University, Seoul, Korea

**Keywords:** Freshwater Oligochaeta, *Nais communis*, complete mitochondrial genome, phylogenetic analysis

## Abstract

The complete mitochondrial genome of *Nais communis* was analyzed using the Illumina Hiseq 2000 platform. The length of the complete mitochondrial genome was 15,685 bp, and the data were submitted to NCBI (MW770354). The genome contained 13 protein-coding genes (PCGs), 2 rRNA genes, 22 tRNA genes, and a putative control region. A phylogenetic tree was constructed based on the sequences of 13 PCGs identified by the maximum-likelihood method. Regardless of the lack of studies on the complete mitochondrial genome of other aquatic oligochaetes, the phylogenetic tree showed *N. communis* to cluster with *Tubifex tubifex* and *Limnodrilus hoffmeisteri* with high support value, and the freshwater oligochaete and earthworm groups to be sister groups.

The genus *Nais* Müller 1774 is a group of oligochaetes within the subfamily Naidinae, family Naididae Ehrenberg, 1828. These species are tiny worms that primarily inhabit freshwater, although some species are adapted to brackish environments (Brinkhurst and Jamieson 1971; Martínez-Ansemil and Prat 1984). *Nais communis* is a common species in this genus and appears to be more common than other *Nais* species in eutrophic waters (Dumnicka 1978; Bonacina et al. 1992; Juget and Lafont 1994; Jung 2011; Lee and Jung 2015). Therefore, as a representative species, *N. communis* is frequently used in ecological studies (Arimoro et al. 2007; Miserendino et al. 2008; Arslan and Mercan 2018). The morphological characteristics most frequently evaluated for differentiation of *Nais* species include the chaeta forms, i.e. ventral, hair chaetae, and needle chaetae. Differences between the chaetae of these species are often subtle and may overlap, leading to taxonomic confusion. This species also forms a species complex with many known cryptic species (Envall et al. 2012). For example, *N. communis* is morphologically similar to *Nais variabilis* Piguet, 1906, which often causes confusion in distinguishing them. The most frequently used morphological character to distinguish between them is the shape of their stomachs. The stomach of *N. variabilis* widens abruptly, whereas that of *N. communis* widens gradually (Brinkhurst and Jamieson 1971; Loden and Harman 1980; Envall et al. 2012). Since this is a minor difference, molecular studies of these two species are required to overcome the ambiguity. However, very few studies have reported the complete mitochondrial genomes of freshwater oligochaetes. In this study, we sequenced the mitogenome of *N. communis* and analyzed its phylogenetic position in the subclass Oligochaeta. We identified our specimen as *N. communis* because its anterior ventral chaetae were thinner and longer than the lower ones, with 4–5 chaetae per bundle. The needles had a clearly visible finely bifid with diverging teeth. Thus, our specimen had similar chaetae to morphotypes A3 and A4, which were regarded as *N. communis* lineages by Envall et al. (2012). Further, it had a gradually widening stomach and could not swim when alive.

The specimen was collected on Jeju Island (Korea) in October 2019 (126° 51′ 21.42″E, 33° 49′ 55.14″N) and preserved in 80% ethanol; the voucher specimen was stored at the National Institute of Biological Resources (no. NIBRIV0000882545). Whole genomic DNA was extracted from posterior body segments of adult specimens using a REPLI-g Mitochondrial DNA Kit (Qiagen, Valencia, CA, USA). Whole-genome sequencing was performed using the Hiseq 2000 platform (Illumina). The mitochondrial genome was constructed using MITObim v1.9.1 (Hahn et al. 2013) and MITOS (Bernt et al. 2013). The sequence was deposited in GenBank (accession number MW770354). One new and 10 published mitochondrial genome sequences downloaded from GenBank, and *Urechis caupo* (Echiuroidea), included as an outgroup, were used for construction of the phylogenetic tree. Subsequently, annotations were performed using Geneious Prime 2019.2.1 (Kearse et al. 2012), and alignment was performed using MUSCLE Alignment. (Thompson et al. 2003). The best selected partitioning schemes and models of evolution were then obtained with ModelFinder (Kalyaanamoorthy et al. 2017), and a GTR + G + I model was identified as the best-fit model for the data. Maximum-likelihood analysis was conducted using PhyML 3.0 (Guindon et al. 2010) with 1000 bootstrap replicates.

The circular mitogenome of *N. communis* was 15,685 bp in size, with an overall base composition of 36.8% for A, 18.1% for C, 13.3% for G, and 31.8% for T. The genome exhibited codon biases with an AT content of 68.6% in protein-coding genes. The mitochondrial genome contained 13 protein-coding genes, two ribosomal RNA genes, and 22 tRNA genes. Of the 13 protein-coding genes, nine (*ATP6*, *ATP8*, *COX1*, *COX2*, *COX3*, *CYTB*, *ND1*, *ND4*, and *ND6*) used ATG as the start codon, two (*ND2*, *ND3*, and *ND4L*) used ATT as the start codon, and one (*ND5*) used ATA as the start codon.

Phylogenetic analysis, based on *N. communis,* of the mitogenomic sequences (13 PCGs) of all 12 species of annelids uploaded to GenBank indicated the relationships among groups within Annelida. Results showed that the newly sequenced species *N. communis* clustered together with *Tubifex tubifex* and *Limnodrillus hoffmeisteri* with high support value, indicating that freshwater oligochaete and earthworm groups are sister groups within Oligochaeta (Figure 1). The relationships ((*N. communis* + *T. tubifex* + *L. hoffmeisteri*) + (*Amynthas triastriatus + Metaphire californica + Metaphire guillelmi + Duplodicodrilus schmardae + Lumbricus rubellus + Drawida japonica*)) *+ Chaetopterus variopedatus + Namalycastis abiuma + Urechis caupo* were supported in Annelida.

**Figure 1.**
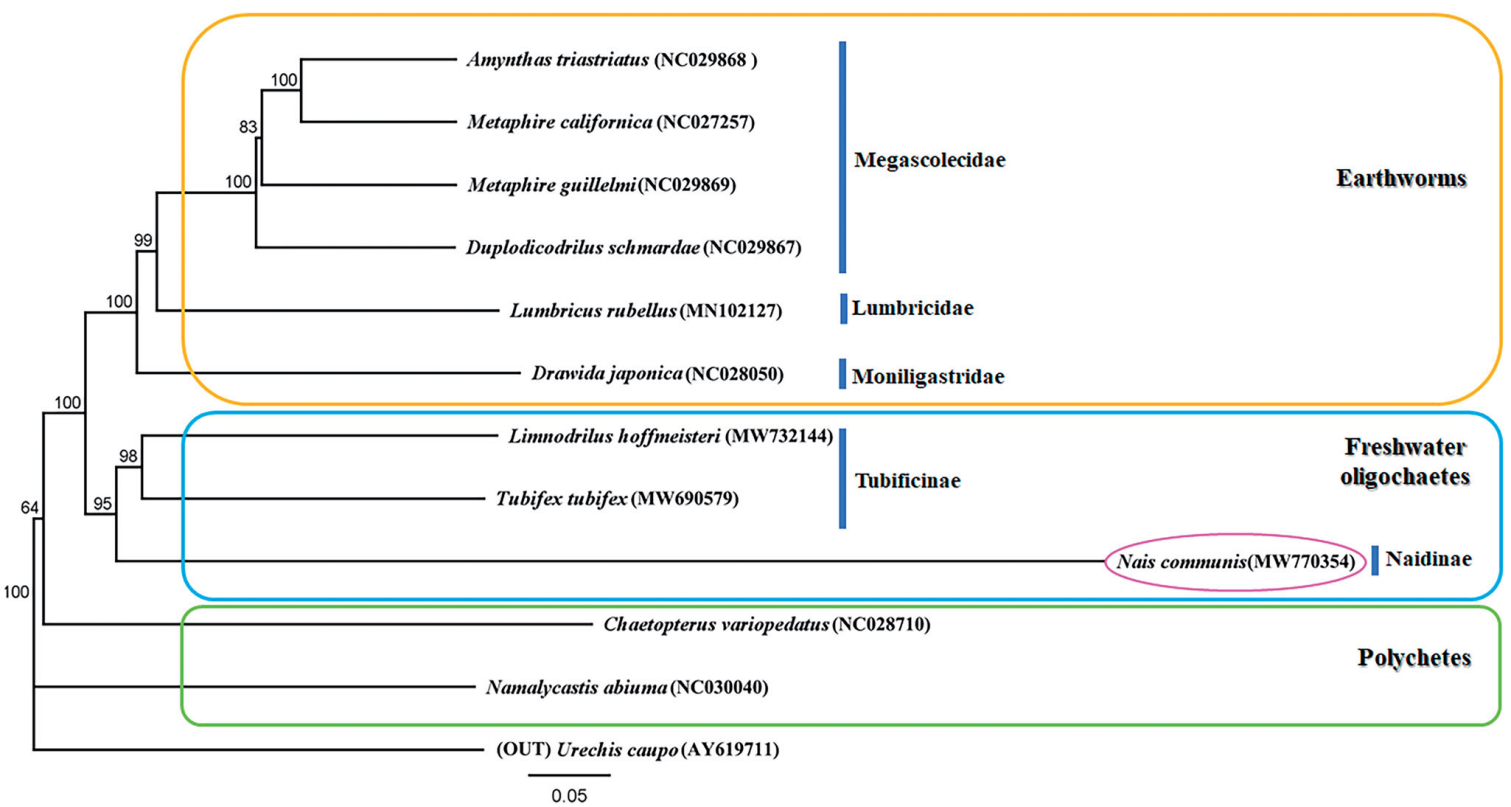
Molecular phylogeny of *N. communis* (MW770354), one species in freshwater oligochaete, 10 species in annelids, and outgroup species based on complete mitogenome. The complete mitogenomes are downloaded from GenBank and the phylogenetic tree is constructed by the Maximum-likelihood method with 1000 bootstrap replicates.

This study further clarified our understanding of the phylogenetic relationships of freshwater oligochaetes.

## Data Availability

The genome sequence data that support the findings of this study are available in GenBank (https://www.ncbi.nlm.nih.gov) under accession no. MW770354. The associated data that support the findings of this study are also openly available in Mendeley Data at http://dx.doi.org/10.17632/7mfvhw5v87.1.
